# Endemic Venezuelan Equine Encephalitis in Northern Peru

**DOI:** 10.3201/eid1005.030634

**Published:** 2004-05

**Authors:** Patricia V. Aguilar, Ivorlyne P. Greene, Lark L. Coffey, Gladys Medina, Abelardo C. Moncayo, Michael Anishchenko, George V. Ludwig, Michael J. Turell, Monica L. O’Guinn, John Lee, Robert B. Tesh, Douglas M. Watts, Kevin L. Russell, Christine Hice, Stephen Yanoviak, Amy C. Morrison, Terry A. Klein, David J. Dohm, Hilda Guzman, Amelia P.A. Travassos da Rosa, Carolina Guevara, Tadeusz Kochel, James Olson, Cesar Cabezas, Scott C. Weaver

**Affiliations:** * University of Texas Medical Branch, Galveston, Texas, USA; † Naval Medical Research Center Detachment, Lima, Peru; ‡ U.S. Army Medical Research Institute of Infectious Diseases, Fort Detrick, Maryland, USA; § University of California, Davis, California, USA; ¶ Instituto Nacional de Salud, Lima, Peru

**Keywords:** Encephalitis Virus, Venezuelan Equine Alphavirus, Arbovirus infections, Endemic diseases, Peru, classification

## Abstract

Since Venezuelan equine encephalitis virus (VEEV) was isolated in Peru in 1942, >70 isolates have been obtained from mosquitoes, humans, and sylvatic mammals primarily in the Amazon region. To investigate genetic relationships among the Peru VEEV isolates and between the Peru isolates and other VEEV strains, a fragment of the PE2 gene was amplified and analyzed by single-stranded conformation polymorphism. Representatives of seven genotypes underwent sequencing and phylogenetic analysis. The results identified four VEE complex lineages that cocirculate in the Amazon region: subtypes ID (Panama and Colombia/Venezuela genotypes), IIIC, and a new, proposed subtype IIID, which was isolated from a febrile human, mosquitoes, and spiny rats. Both ID lineages and the IIID subtype are associated with febrile human illness. Most of the subtype ID isolates belonged to the Panama genotype, but the Colombia/Venezuela genotype, which is phylogenetically related to epizootic strains, also continues to circulate in the Amazon basin.

Venezuelan equine encephalitis virus (VEEV) is an emerging mosquito-borne RNA virus in the family *Togaviridae*, genus *Alphavirus*. Since its isolation in 1938, many equine epizootics and epidemics have been reported in Colombia, Venezuela, Trinidad, Peru, Ecuador, Mexico, and the United States, and other countries (*1*–*5*); the number of reports of human and equine cases have increased during recent years (*6*–*8*). At least 13 distinct subtypes and varieties, including several different species, make up the VEE complex (Table 1). Only subtype I varieties A, B, and C have caused major outbreaks involving hundreds of thousands of equine and human cases. Subtypes II through VI and subtype I varieties D, E, and F are enzootic, equine-avirulent strains not associated with major equine outbreaks or epidemics, although they do cause human illness, which can be fatal (*9*,*10*).

**Table 1:** Classification of the Venezuelan equine encephalitis (VEEV) complex of alphaviruses^a^

Subtype	Variety	Species	Transmission pattern	Equine virulence^b^	Location	Vector
I	AB	VEEV	Epizootic	virulent	Central, South, North America	Mammalophilic mosquitoes
	C	VEEV	Epizootic	virulent	South America	mammalophilic mosquitoes
	D	VEEV	Enzootic	avirulent	Central, South America	*Culex (Mel.) ocossa, panocossa, pedroi, adamesi, vomerifer*
	E	VEEV	Enzootic	variable	Central America, Mexico	*Cx. (Mel.) taeniopus*
	F	Mosso das Pedras	Enzootic	unknown	Brazil	unknown
II		Everglades	Enzootic	avirulent	Southern Florida	*Cx (Mel.) cedecei*
III	A	Mucambo	Enzootic	avirulent	South America	*Cx (Mel.) portesi*
	B	Tonate	Enzootic	unknown	South, North America	Unknown (Tonate), bug (Bijou Bridge strains from N. America)
	C		Enzootic	unknown	Peru	Unknown
IV		Pixuna	Enzootic	unknown	Brazil	Unknown
V		Cabassou V	Enzootic	unknown	French Guiana	Unknown
VI		Rio Negro	Enzootic	unknown	Argentina	Unknown

^a^Source: The Arboviruses: Epidemiology and Ecology (9). ^b^Virulent strains produce high viremia titers and generally >50% death rates in horses experimentally infected.

In Peru, VEEV was first isolated in the 1940s, when subtype IAB strains caused equine epizootics and epidemics along the Pacific coast (*7*,*8*). Field investigations were later conducted to determine the origin of the IAB strains. Initial ecologic studies in the Amazon region of northeastern Peru (*11*,*12*) yielded 11 VEE complex isolates from mosquitoes and sentinel hamsters during 1970 and 1971 in Quistococha, near Iquitos (Figure 1). Antigenic analyses identified 10 isolates as subtype ID VEEV (*11*) and one strain as subtype IIIC in the VEE complex (*12*,*13*). Because these viruses were isolated only from sentinel hamsters and mosquitoes, whether they were human pathogens remained unclear. Not until 1993 through 1995, when VEEV subtype ID was isolated from persons with febrile illness in the Amazon region of Peru (*4*,*14*), was there clear evidence that VEEV causes human disease in this region. Two subtype ID strains were isolated from patients in Pantoja in 1994, and 10 other subtype ID strains were obtained from febrile patients in and around the city of Iquitos from 1993 to 1995.

**Figure 1:**
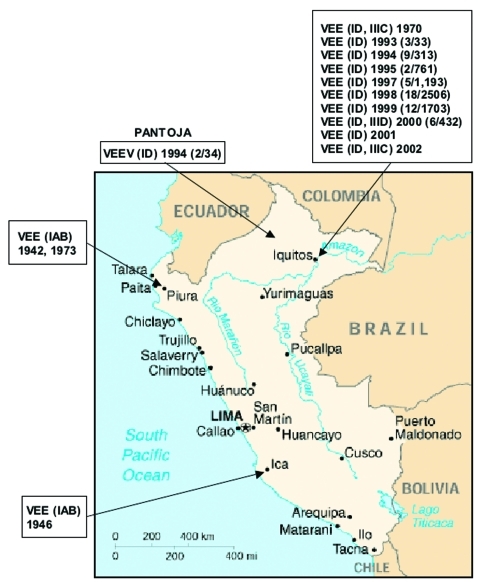
Map of Peru showing the geographic distribution of the Venezuelan equine encephalitis virus (VEEV) complex isolates included in the study. Numbers in parenthesis indicate the number of isolates compared to the total number of febrile cases during the year. ID, IAB, IIIC, IIID correspond to VEEV subtypes isolated during the indicated year.

Nucleotide sequences and phylogenetic analyses recently performed on Peruvian VEEV isolates indicated that the 1970s VEEV subtype ID mosquito isolates and the 1994 Pantoja human isolates belong to the Colombia/Venezuela ID genotype, whereas the 1993–1995 Iquitos human isolates belonged to the Panama ID genotype (*9*). Lack of detection of the Colombia/Venezuela genotype in the Iquitos area during 1993 through 1995, when the Panama genotype was repeatedly isolated, suggested that the Colombia/Venezuela genotype had been replaced by virus strains from Panama. Because the Colombia/Venezuela VEEV genotype is believed to give rise to epizootic viruses through mutations of the E2 envelope glycoprotein (*15*–*18*), disappearance of this genotype from the Iquitos area could have important public health implications.

Since 1995, 75 VEEV strains have been isolated from mosquitoes, humans, and sentinel hamsters in Peru. We determined the genetic relationships among strains isolated from 1995 through 2002 and compared them with other VEEV strains isolated from the Americas. Our results indicate that the VEEV subtype ID Colombia/Venezuela genotype continues to circulate in Iquitos but may infect people at a lower rate than the Panama genotype. We also demonstrated that a recent subtype III isolate is genetically distinct from the subtype IIIC strain isolated in 1971 (*12*). This strain, which we propose as subtype IIID in the VEE complex, and both subtype ID genotypes cause human febrile disease in the Iquitos area.

## Methods

### Study Sites

VEEV isolates were obtained from several locations in Peru. The main study site was centered around Iquitos, a city of 300,000 people on the Amazon River in the Department of Loreto (Figure 1). The major occupations of the inhabitants are housekeeping, teaching, military work, agriculture, fishing, and tourism. The climate is tropical, with a mean annual temperature of 27.5°C and mean annual precipitation of 2.7 m (*4*).

### Virus Isolates

The VEE complex isolates included in this study are shown in Table 2. These viruses were provided by the University of Texas Medical Branch World Health Organization Collaborating Reference Center for Arboviruses, the United States Army Medical Research Institute of Infectious Diseases, and the U.S. Naval Medical Research Center Detachment, Lima, Peru (NMRCD). Most of the viruses were isolated in Vero cells from serum of febrile patients, sentinel hamsters, and mosquitoes. These viruses were passaged once in Vero cells, and supernatant fluid was stored at –70°C for subsequent extraction of viral RNA.

**Table 2:** Venezuelan equine encephalitis complex virus isolates included in this study

Subtype	Code	Location	Year	Host	Signs and symptoms^a^
ID	IQT0988	Sanidad EP/Vargas Guerra, Iquitos	1993	Human	F, A
ID	IQT1015	Sanidad EP/Vargas Guerra, Iquitos	1993	Human	F, H, C
ID	IQT1026	Sanidad EP/Vargas Guerra, Iquitos	1994	Human	F, H, E, B, D, C, G, N, S
ID	DEI5191	Pantoja, Iquitos	1994	Human	F, C, H, B
ID	DEI5193	Pantoja, Iquitos	1994	Human	F, C, I, H, B, N, V
ID	IQT1042	Dir. Reg Salud de Loreto, Iquitos	1994	Human	F, H, B, V
ID	IQT1071	Pedrelor, Iquitos	1994	Human	F, H, B, E, A, I, C
ID	IQT1081	Pedrelor, Iquitos	1994	Human	F, H, B, E, D, R, C
ID	IQT1085	Hospital Regional Amazonas, Iquitos	1994	Human	F, H, B, D, G, N, S
ID	IQT1098	Sanidad EP/Vargas Guerra, Iquitos	1994	Human	F, H, E, B, D, C, S
ID	IQT1101	Pedrelor, Iquitos	1994	Human	F, H, E, B, D, V, C, G
ID	IQT1120	Sanidad EP/Vargas Guerra, Iquitos	1994	Human	F, H, B, D, V, C
ID	IQT1724	Pedrelor, Iquitos	1995	Human	F, H, E, B, D, V, C, I, G
ID	IQT1735	Pedrelor, Iquitos	1995	Human	F, H, B, E, D, R, V, C
ID	IQT3745	Sanidad EP/Vargas Guerra, Iquitos	1997	Human	F, H, E, B, D, G
ID	IQT3971	San Antonio, Iquitos	1997	Human	F, H, E, B, D, V, I, C
ID	IQT4091	Bellavista, Iquitos	1997	Human	F, B, D, V, I, M, V, S, J
ID	IQT4177	Sanidad EP/Vargas Guerra, Iquitos	1997	Human	F, H, E, B, D, V, C, G
ID	IQT4191	Bellavista, Iquitos	1997	Human	F, H, E, B, D, C, S
ID	PC28	Iquitos	1997	Rodent (*Proechimys* spp)	
III	PC254	Iquitos	1997	Rodent (*Proechimys* spp)	
III	PC256	Iquitos	1997	Rodent (*Proechimys* spp)	
ID	IQT5798	San Juan, Iquitos	1998	Human	F, H, E, B, D, C
ID	IQT5831	Cardoso, Iquitos	1998	Human	F, H, E, B, D, C
ID	IQT5876	San Juan, Iquitos	1998	Human	F, H, E, B, D, V, C
ID	IQT5885	Moronacocha Iquitos	1998	Human	F, H, E, B, D, V, G, T
ID	IQT6088	9 de Octubre, Iquitos	1998	Human	F, H, E, B, D, V, I, C, G
ID	IQT6119	San Juan, Iquitos	1998	Human	F, H, E, B, D, R, V, I, C
ID	IQT6415	San Juan, Iquitos	1998	Human	F, H, E, B, D, V, I, C
ID	IQT6486	9 de Octubre, Iquitos	1998	Human	F, H, E, B, D, V
ID	IQT6674	Tupac Amaru, Iquitos	1998	Human	F, H, E, B, D, C
ID	IQT6712	Hospital Apoyo, Iquitos	1998	Human	F, H, E, B, D, V, S
ID	IQT6937	NMRCD, Iquitos	1998	Human	F, H, E, B, D, V, S
ID	IQT7057	San Juan, Iquitos	1998	Human	F, H, E, D, V, C
ID	IQT7060	San Juan, Iquitos	1998	Human	F, H, E, B, D, C
ID	IQT7327	Tupac Amaru, Iquitos	1998	Human	F, H, E, B, D, V, C, G
ID	IQT7460	CIA Petrolera, Iquitos	1998	Human	F, H, B, C
ID	IQT7988	Belen, Iquitos	1998	Human	F, H, B, D, V, C, G, S
ID	IQT8131	Belen, Iquitos	1998	Human	F, H, E, B, D, R, V, C, D
ID	IQT8558	San Juan, Iquitos	1998	Human	F, H, B, D, V, I, C, G
ID	PE30609	Iquitos	1998	Mosquito	
III	PE407660	Iquitos	1998	Mosquito	
III	PE409040	Iquitos	1998	Mosquito	
III	PE409100	Iquitos	1998	Mosquito	
ID	IQU0465	San Antonio, Iquitos	1999	Human	F, H, E, B, D, R, C
ID	IQU0664	San Antonio, Iquitos	1999	Human	F, H, E, B, D, C
ID	IQU0890	6 de octubre, Iquitos	1999	Human	F, H, B, E, D, V, C
ID	IQU0953	6 de octubre, Iquitos	1999	Human	F, H, B, E, C
ID	IQU1050	Belen, Iquitos	1999	Human	F, H, B, E, V, C
ID	IQU1106	San Antonio, Iquitos	1999	Human	F, H, E, B, A, V, C
ID	IQU1217	Belen, Iquitos	1999	Human	F, H, E, B, D, V, I, C, G
ID	IQU1279	Belen, Iquitos	1999	Human	F, H, E, B, V, C, G
ID	IQU1282	Cardozo, Iquitos	1999	Human	F, H, E, B, D, C
ID	IQU1318	Tupac Amaru, Iquitos	1999	Human	F, H, E, B, D, V, C
ID	IQU1341	Zungarococha, Iquitos	1999	Human	F, H, E, B, D, R, V, C
ID	IQU1402	Cardozo, Iquitos	1999	Human	F, H, E, B, A, V, C, G, S
ID	IQU1718	San Antonio, Iquitos	1999	Human	F, H, E, B, D, I, C
III	FSL0190	San Juan, Iquitos	2000	Human	F, C, L
ID	FSL0201	San Juan, Iquitos	2000	Human	F, C, L, D, B, A
ID	FSL0205	San Juan, Iquitos	2000	Human	F, C, L, D, B, A
ID	FSL0240	San Juan, Iquitos	2000	Human	F, C, L, D, A, I, V, J, G
ID	FSL0252	San Juan, Iquitos	2000	Human	F, C, L, D, B, A
ID	IQU3026	Hospital Apoyo, Iquitos	2000	Human	F, H, E, B, D, V, C, S
ID	FSL0507	Hospital Militar, Iquitos	2001	Human	F, H
IIIC	54-001	Iquitos	2002	Hamster	

^a^A, abdominal pain; H, headache; F, fever; C, chills; E, eye pain; B, body pain; D, arthralgia; G, cough; N, nasal congestion; S, sore throat; I, diarrhea; V, vomits; R, rash; M, melena; J, jaundice; T, hematuria; L, malaise.

### Extraction of Viral RNA and cDNA Synthesis

Viral RNA was extracted from cell culture media as described elsewhere (*19*). Briefly, 250 μL of infected cell culture supernatant was mixed with 750 μL of Trizol LS (Gibco BRL, Bethesda, MD), and RNA was extracted following the manufacturer’s protocol. For cDNA synthesis, 5 μL of the extracted RNA was mixed with 1 μmol of reverse primer V9207B (Table 3), 1X First Strand Buffer (Gibco BRL, Bethesda, MD), 1 mmol deoxynocleoside triphosphate (dNTPs), 80U RNAsin (Promega, Madison, WI), and 200 U SuperScript II Reverse Transcriptase (Gibco BRL, Bethesda, MD), and then incubated at 42°C for 1 h.

**Table 3:** Oligonucleotides used for polymerase chain reaction amplification and sequencing analysis

Primer (genetic sense)	Sequence (5′→3′)
V8369(+)	GAGAACTGCGAGCAATGGTCA
V9207B(-)	TRCACTGGCTGAACTGTT
V9257B(-) V8659(+)	TACACCCAYTTRTCRTTCTG AATTGAGGCAGTGAAGAGCGAC
V8953(-)	CTGCCTACAGGATTAAAT
E/V 7514(+)	ACYCTCTACGGCTRACCTRA
VIIID 10471(-) α10247A(+)	CCTTCCGGTCGAACGGGGTCC TACCCNTTYATGTGGG
Mlu-T25 (-)	TTACGAATTCACGCGTTTTTTTTTTTTTTTTTTTTTTTTT

### PCR Amplification

Primers used for the polymerase chain reaction (PCR) and sequencing are listed in Table 3. For amplification of the N-terminus of the PE2 envelope glycoprotein precursor gene, primers V8369(+) and V9207B(-) were used, and the resulting product was sequenced with primers V8659(+) and V8953(-) (*19*). PCRs included 2.5 U of Taq polymerase (Promega, Madison, WI), 1X Promega Taq buffer, 300 nmol of each primer, 1 mmol MgCl_2_, 0.2 mmol dNTPs, and 10 μL of the cDNA reaction; 30 amplification cycles included heat-denaturation at 95°C for 30 s, primer annealing at 48°C for 30 s, and extension at 72°C for 1 min. A final extension of 10 min was used to ensure complete double-stranded DNA synthesis. In the case of the proposed subtype IIID strains*,* the reverse primer used for cDNA synthesis and PCR was V9257B(-). In some cases, PE2 amplicons were not obtained, and those isolates were amplified by using alphavirus-specific nsP1 primers described previously (*20*). The complete 26S gene of one subtype IIID strain (human isolate FSL190) was amplified and sequenced for further comparison with other VEE complex subtypes; two overlapping PCR amplicons were obtained by using primer pairs E/V 7514(+)/VIIID 10471(-), and α10247A(+)/Mlu-T25(-), and sequenced by gene walking.

### Single-Stranded Conformation Polymorphism (SSCP)

SSCP analysis was performed as previously described (*16*). PCR products were purified with the Qiaquick gel extraction kit (Qiagen, Valencia, CA), and 2 μL of the purified PCR amplicon DNA was mixed with 8 μL of SSCP loading buffer (95% formamide, 0.05% bromophenol blue, 0.05% xylene cyanol). To denature the DNA, the reaction was heated to 95°C for 5 min and immediately cooled on ice, loaded onto an 8% polyacrylamide gel, and electrophoresed in 1 x Tris-borate-EDTA buffer at room temperature for 20 h at 8 mA. The single-stranded DNA products were visualized with silver staining (*21*). Single-strand conformational polymorphism genotypes were determined by comparing the migration patterns of the double-stranded DNA of the various isolates with one another and against a standard DNA ladder. Unique migration patterns were designated as distinct SSCP genotypes.

### Sequencing and Phylogenetic Analysis

Two or three representatives of each SSCP genotype were selected randomly for sequencing and phylogenetic analysis. Most PCR products were sequenced directly with an Applied Biosystems (Foster City, CA) Prism automated DNA sequencing kit according to the manufacturer’s protocol. In some cases, PCR products were cloned into the PCR II vector (Invitrogen, Carlsbad, CA), and at least two clones were sequenced by using vector-specific primers. Sequences were aligned by using the Clustal program in the MacVector (Oxford Molecular Group, Campbell, CA) software package, and phylogenetic analyses were conducted by using maximum parsimony, neighbor-joining, and maximum likelihood programs implemented in the PAUP 4.0 software (*22*). For the neighbor joining analysis, the HKY85 distance formula was used. Bootstrap analyses were conducted with 1,000 replicates to place confidence values on groupings within trees (*23*).

### Plaque Reduction Neutralization Tests

To obtain immune sera against VEEV subtypes IIIA, IIIB, IIIC and IIID, cotton rats (*Sigmodon hispidus*, Harlan, Indianapolis, IN) were infected subcutaneously with 1,000 PFUs of virus. Three weeks after injection, the animals were bled for antibody testing using plaque reduction neutralization tests (PRNT) with Vero cells (*24*). Viruses were tested against all antibody preparations, and the endpoint titers were defined as the reciprocal of the highest dilution inhibiting >80% of approximately 100 PFU of virus.

## Results

### Distribution and Characterization of Human VEEV Cases in Iquitos

**Figure 2:**
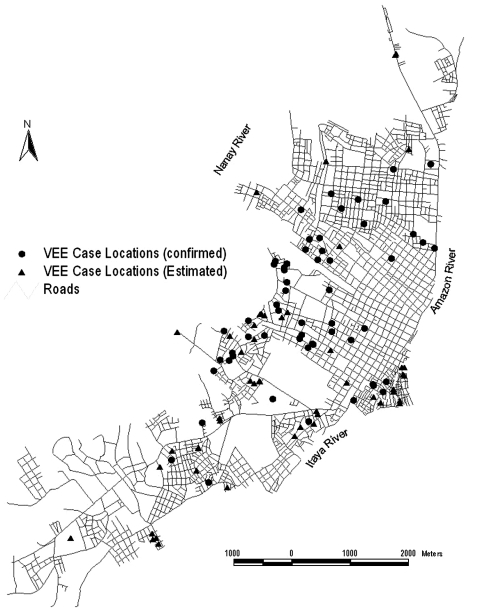
Map of Iquitos showing the locations of human Venezuelan equine encephalitis virus cases in the city.

Studies to identify the cause of human febrile illness in the Amazon region of Peru since 1995 (Watts et al., unpub. data) identified at least 183 cases of VEEV on the basis of immunoglobulin (Ig) M antibody detection, virus isolation, or both. The VEEV cases in the Iquitos area were localized into two major clusters: the region surrounding Lake Morona Cocha, which typically floods during the rainy season, and cases near the Itaya and the Amazon rivers (Figure 2). Cases were also observed to the north and southwest of Iquitos. Most patients with VEEV did not report travel outside their home region, suggesting that many infections occurred in the city of Iquitos. Common signs and symptoms of VEEV infection included fever, headache, chills, malaise, diarrhea, vomiting, arthralgia, and abdominal pain. Clinical information obtained by NMRCD provided no indication of neurologic disease, and no deaths were reported.

### Sequencing and Phylogenetic Analysis

An 856-bp fragment from the N terminus of the PE2 gene was obtained for most of the VEEV subtype ID isolates using primers V8369(+)/V9207(-). This region was chosen because it has been used previously in similar phylogenetic analyses, resulting in a large database that allows for genetic comparison with new isolates (*4**,**9**,**25*). In addition, the fragment sequenced contains the N-terminal portion of the E2 glycoprotein gene, which has been shown to undergo critical amino acid substitutions associated with epizootic VEEV emergence (*18*).

To screen the amplified isolates for genetic differences and to eliminate the cost and effort in sequencing each virus isolate, SSCP analyses were performed as previously described (*19*). At least 2–3 representatives of each SSCP genotype were selected at random for sequence and phylogenetic analysis. Some isolates could not be amplified with primers V8369(+)/V9207B(-), and later phylogenetic analyses indicated that they corresponded mainly to subtypes other than ID. For these isolates, primer V9257(-) was substituted for V9207B(-) in the cDNA syntheses and PCR amplifications. In addition, alphavirus consensus primers (*20*) that amplify a portion of the nsP1 gene were used to confirm the identity of these isolates and to look for evidence of recombination within the VEE complex (data not shown).

**Figure 3:**
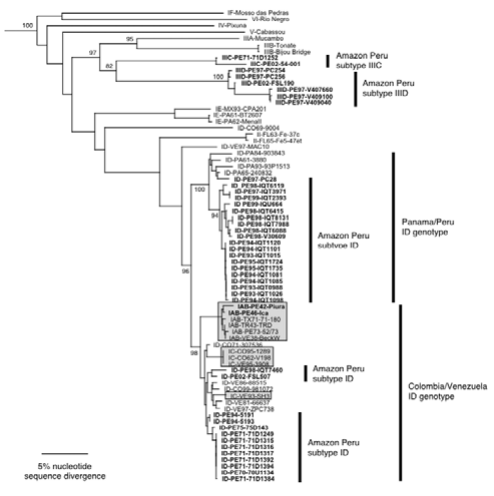
Phylogenetic tree of the Venezuelan equine encephalitis virus (VEEV) complex derived from partial PE2 gene sequences of Peruvian VEEV isolates and homologous sequences published previously, using the neighbor joining program implemented in PAUP 4.0 (*22*). The tree was rooted using an outgroup comprised of four major lineages of Eastern Equine Encephalitis virus (*26*). Virus strains are labeled by VEE complex subtype, abbreviated country (FL=Florida, USA) and year of isolation, followed by strain designation. Numbers indicate bootstrap values for clades to the right.

Phylogenetic analyses performed using maximum parsimony, neighbor-joining, and distance-matrix methods generated similar tree topologies. The neighbor-joining tree based on the PE2 gene (Figure 3) showed that the newly sequenced VEEV Peruvian strains grouped into four major clades: the subtype ID Colombia/Venezuela genotype, the ID Panama/Peru genotype, subtype IIIC isolated in 1971 in the Iquitos area of Peru from a mosquito, and a newly identified group of viruses that fell within subtype III but were distinct from IIIC, which we propose as a new genetic subtype IIID.

The greatest number of human isolates from Peru fell into VEEV subtype ID. In agreement with previous studies (*9*), genetic analysis of the ID strains delineated two distinct genotypes that differ by approximately 5% in their nucleotide sequences: the Colombia/Venezuela genotype, which is believed to have generated the epizootic IAB and IC viruses (*15*–*18*), and the Panama/Peru genotype. The Colombia/Venezuela ID genotype was represented by isolates in Peru during the 1970s and two isolates made from Pantoja in 1994. Previous data suggested that this genotype was no longer circulating in Iquitos and had been replaced by the Panama genotype; however, the evidence that these viruses continued to circulate in Iquitos was supported by human isolates from 1998 and 2002, which grouped into the Colombia/Venezuela clade with strong bootstrap support (Figure 3). However, most of the Peruvian VEEV isolates from the Iquitos region grouped into the ID Panama/Peru genotype and were obtained from humans, rodents, and mosquitoes.

The VEE complex subtype IIIC strain, first isolated in 1971 from mosquitoes collected near Iquitos (*12*,*13*), was isolated again in 2002 from a sentinel hamster in the Amazon region. Several other isolates from mosquitoes, humans, and rodents also grouped into subtype III (88% bootstrap support) but were quite distinct from the subtype IIIC isolates (18% nucleotide sequence, 8% amino acid sequence divergence in the PE2 protein genes). These levels of divergence are equal to or greater than those of other VEE complex antigenic subtypes, so we propose the designation of subtype IIID for these strains.

**Figure 4:**
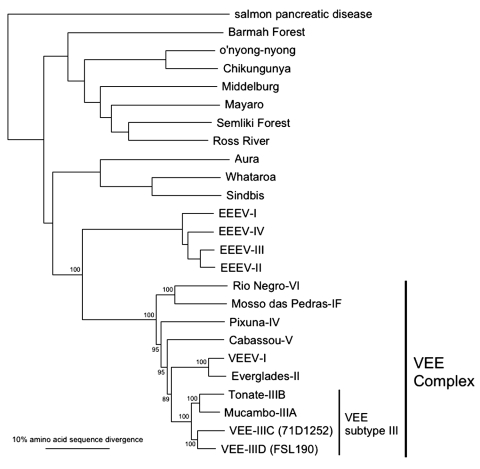
Unrooted phylogenetic tree of the Venezuelan equine encephalitis virus (VEEV) complex and other representative alphaviruses derived from complete structural polyprotein sequences using the neighbor joining program implemented in PAUP 4.0 (*22*). Viruses are labeled by species according to the International Committee for Taxonomy of Viruses (*27*). VEEV subtype IIIC and IIID strain names are in parentheses. Numbers indicate bootstrap values for clades to the right.

To further document these relationships within subtype III and to investigate possible recombination, phylogenetic trees on the basis of the nsP1 gene were also constructed. The nsP1 trees did not show any evidence of recombination and also supported the sister-group relationship between subtype IIIC and the related IIID isolates (data not shown). To further genetically characterize this new subtype III strains, trees based on the complete 26S structural gene sequences were generated. All trees, including the neighbor-joining tree shown in Figure 4, confirmed the close relationship between the IIIC and IIID subtypes, as well as their level of divergence.

### Antigenic Characterization of Proposed Subtype IIID

To characterize antigenically the putative new VEEV subtype IIID, PRNTs were performed to determine the antigenic relationships among the subtype III viruses (Table 4). Mucambo virus (subtype IIIA) versus Tonate virus (subtype IIIB) showed a fourfold difference in only one direction, which, based on the traditional serologic classification criteria (*26*), indicates that these are virus subtypes. When subtype IIIC was compared with the IIIA and IIIB subtypes, a fourfold or greater difference was observed in both directions, indicating that IIIC is a different virus. However, only a twofold difference existed in endpoint titers between subtype IIIC and the new IIID, which indicated that they are not distinct subtypes according to traditional antigenic criteria (*28*).

**Table 4:** Results of the plaque reduction neutralization tests between members of Venezuelan equine encephalitis subtype III^a^

	Antiserum (subtype/variety)			
Virus strain (subtype/variety)	Mucambo (IIIA)	Tonate (IIIB)	71D1252 (IIIC)	PE407660 (IIID)
Mucambo (IIIA)	1	2	16	8
Tonate (IIIB)	4	1	>160	8
71D1252 (IIIC)	>32	4	1	2
PE4.07660 (IIID)	8	16	2	1

^a^Numbers indicate ratios of homologous to heterologous reciprocal titers.

## Discussion

VEEV is considered an emerging human pathogen in Latin America because a resurgence of VEE disease has occurred in Mexico and South America during the past decade (*8*). Our results indicate that VEEV is also endemic in Peru, with cases occurring regularly in the Iquitos area from 1993 through 2002. Analysis of a larger number of VEEV isolates allowed us to generate a more accurate and complete description of VEE complex circulating in the Amazon Basin of Peru than was possible previously (*9*). Most human isolates belong to subtype ID, and phylogenetic analyses distinguish two distinct ID genotypes: the Colombia/Venezuela genotype, which is currently circulating in Peru, contrary to previous suggestions (*9*), and the Panama/Peru genotype, which represents most human isolates. Several hypotheses explain the apparently higher rate of human infection in the Iquitos area by the ID Panama/Peru genotype: 1) viruses from the Colombia/Venezuela genotype are not transmitted efficiently to humans compared to those in the Panama-Peru genotype; 2) the Panama/Peru genotype circulates at higher levels in urban areas such as Iquitos, resulting in more infections; 3) the Panama/Peru genotype causes more severe disease, with more patients visiting clinics and participating in the NMRCD febrile illness study, resulting in more virus isolations and; 4) the Panama/Peru genotype produces higher titer human viremia, resulting in more human isolates. Further ecologic, epidemiologic, and entomologic studies are needed to test these hypotheses. The mosquito vector(s) that transmit VEEV to humans in Peru are unknown. VEEV has been isolated from *Culex* (*Melanoconion*) spp. mosquitoes in Peru, known vectors of enzootic VEEV in many other locations. Several different species (*Culex* [*Mel*.] *gnomatos,* *Cx*. [*Mel*.] *pedroi*, *Cx*. [*Mel*.] *vomerifer*, and *Psorophora albigenu*) are competent laboratory vectors of the ID viruses currently circulating in the Iquitos area (*29*,*30*). Further studies are required to determine whether these mosquitoes, other species, or both transmit VEEV to humans in Peru. This information will be important in understanding human exposure and infection with these different VEE complex strains.

Epidemiologic information available from 1995 through 2002 suggests that many VEE cases occurred within the city of Iquitos. Interviews indicated that many patients had not traveled during the time of probable infection, suggesting urban transmission. Studies carried out in other VEEV-enzootic areas indicate that transmission is confined to rural and forest habitats (*30*–*32*). Whether the viruses have adapted to infect peridomestic mosquitoes or whether Amazonian deforestation and urbanization is increasing the risk for VEEV transmission to humans through changes in *Cx. Melanoconion* spp. host preference and larval habitats remains to be determined.

### Subtype III VEE Complex Strains

Before the strain reported herein, the only isolate of subtype IIIC was obtained in 1971 from a pool of mosquitoes collected near Iquitos (*12*,*13*). Although epidemiologic and ecologic studies were conducted in the same area between 1971 and 1995, no evidence was obtained that the IIIC virus was still circulating in the Peruvian Amazon until we isolated it from a sentinel hamster in 2002. The 2002 IIIC isolate did not produce fatal disease in the sentinel animal nor in other hamsters infected experimentally in our laboratory (data not shown). The failure to isolate the subtype IIIC virus from febrile patients in Iquitos suggests that it is not transmitted readily to humans, that the viremia generated in humans is relatively low and usually undetectable by virus isolation, or that the virus simply does not cause clinical human disease.

We propose that the newly identified subtype III strain be defined as a new genetic variety: subtype III, variety D in the VEE complex. These strains do not represent a new antigenic subtype (*28*) because they exhibit a twofold difference in homologous versus heterologous PRNT antibody titers when compared to the IIIC strain. Arbovirus varieties are described as “isolates differentiable only by the application of special tests or reagents [kinetic hemagglutination inhibition (HI), monoclonal antibody assays, etc.] (*28*).” We propose that this definition of special tests for variety assignment be extended to include sequence and phylogenetic differentiation of major lineages, such as IIIC versus the proposed IIID genetic variety. These lineages are equivalent in genetic divergence to other VEEV varieties defined previously based on serologic reactions using kinetic HI (*8*,*33*).

Both subtype III variants isolated in the Iquitos area are antigenically distinct from Mucambo (IIIA) and Tonate (IIIB) viruses, based on greater than fourfold endpoint antibody titer differences in both directions. The variants are also quite distinct genetically from Mucambo and Tonate viruses (23% nucleotide and 14% amino acid sequence divergence in the structural protein genes) and appear to be geographically distinct as well. We do not have enough information available to know whether the IIIA, IIIB, IIIC, and IIID variants share vectors or reservoir hosts, although IIIA, IIIC, and IIID probably infect *Proechimys* spp., and Tonate virus (IIIB) may use birds as its reservoir hosts (*34*).

The newly identified subtype IIID strain was isolated from spiny rats (*Proechimys* spp.), *Culex* (*Melanoconion*) spp. mosquitoes and from a patient with fever, chills, and malaise. The symptoms are typical of human VEEV (subtype I) infection. Using diagnoses based on virus isolation and serology, Watts et al. (*4*,*14*) previously reported that VEEV was responsible for at least 3% of febrile illnesses in the city of Iquitos in the Amazon basin of Peru. Subtype ID was the thought to be the cause of these human VEEV cases, including those diagnosed only by serologic findings. However, our study demonstrated that both VEEV subtype ID and the newly recognized subtype IIID are responsible for human illness in the Peruvian Amazon basin. Whether subtype IIID was responsible for any other of the 183 human cases diagnosed serologically in Peru since 1995 is unclear. More detailed studies to retrospectively examine serum samples from these cases are required to evaluate this possibility.

### Human Disease and Virulence of Enzootic VEEV Strains

In Peru, human VEE does not appear to result in neurologic manifestations, and fatal human disease has never been reported. In contrast, during recent VEEV epidemics with subtype IC strains in Colombia and Venezuela, an estimated 3,000 cases with neurologic complications and 300 fatal cases (*5*,*35*) were reported. Overall, human death rates have generally been estimated at approximately 0.5% during these epidemics, with most of the neurologic disease and fatal cases reported in children. Most of the human VEEV cases we studied in Peru occurred in adults (94.6%), which suggests an occupational exposure or possibly an age-biased recruitment into the NMRCD febrile illness study. The lack of any evidence for neurologic disease in any of the NMRCD cases studied from 1994 to 2003 (D. Watts, unpub. data) suggests a possible difference in virulence compared with epizootic IAB and IC strains. Because we characterized only 183 VEEV cases, and only 10 of these were children, whether a virulence difference exists between enzootic subtype ID and epizootic VEEV strains is impossible to determine with any statistical certainty. Strains of the Panama-Peru genotype of subtype ID are known to have caused fatal human disease (*10*).
